# Optimization of the Extraction of Chitosan and Fish Gelatin from Fishery Waste and Their Antimicrobial Potential as Active Biopolymers

**DOI:** 10.3390/gels9030254

**Published:** 2023-03-22

**Authors:** Javier Rocha-Pimienta, Bruno Navajas-Preciado, Carmen Barraso-Gil, Sara Martillanes, Jonathan Delgado-Adámez

**Affiliations:** Scientific and Technological Research Center of Extremadura (CICYTEX), Technological Agri-Food Institute of Extremadura (INTAEX), Avda. Adolfo Suárez s/n, 06071 Badajoz, Spain

**Keywords:** revalorization, circular economy, active packaging, tench, prawn, biomaterials, industrial transfer

## Abstract

Fishery residues are abundant raw materials that also provide numerous metabolites with high added value. Their classic valorization includes energy recovery, composting, animal feed, and direct deposits in landfills or oceans along with the environmental impacts that this entails. However, through extraction processes, they can be transformed into new compounds with high added value, offering a more sustainable solution. The aim of this study was to optimize the extraction process of chitosan and fish gelatin from fishery waste and their revalorization as active biopolymers. We successfully optimized the chitosan extraction process, achieving a yield of 20.45% and a deacetylation degree of 69.25%. For the fish gelatin extraction process, yields of 11.82% for the skin and 2.31% for the bone residues were achieved. In addition, it was demonstrated that simple purification steps using activated carbon improve the gelatin’s quality significantly. Finally, biopolymers based on fish gelatin and chitosan showed excellent bactericidal capabilities against *Escherichia coli* and *Listeria innocua*. For this reason, these active biopolymers can stop or decrease bacterial growth in their potential food packaging applications. In view of the low technological transfer and the lack of information about the revalorization of fishery waste, this work offers extraction conditions with good yields that can be easily implemented in the existing industrial fabric, reducing costs and supporting the economic development of the fish processing sector and the creation of value from its waste.

## 1. Introduction

The fish processing industry is responsible for a large amount of waste, including skins, bones, fins, scales, and swim bladders, which constitute 36% (and possibly represent up to 60%) of the raw material mass. There is a significant trend towards the efficient utilization of aquatic resources, since environmental rules regulating the dumping of fish waste are expanding [1]. The reuse of underutilized or discarded marine material might constitute a sustainable approach for the implementation of a circular bioeconomy with the creation of materials with high added value, considering the rising interest in the circular economy [2]. Taking into consideration the facts that they are rich sources of molecules with added value, such as enzymes, bioactive peptides, and biopolymers, and that these compounds have several potential uses in a variety of fields, numerous studies have been published to examine their potential uses [3,4]. Three of the main byproducts of the sector are crustacean shells and fish bones and skins, wastes with enormous potential to produce natural biopolymers of high interest for a multitude of industries.

**Figure 1:**
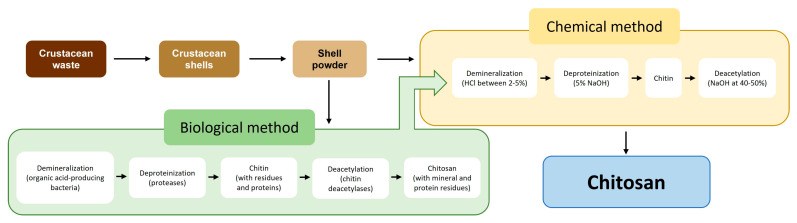
Methods of chitosan extraction [10].

The second most prevalent biopolymer in nature, after cellulose, is chitin, a natural polysaccharide found in the exoskeletons of insects, crustaceans, and fungi [5]. Chitosan is a fiber-like substance and a homopolymer of ß-(1→4)-linked N-acetyl-D-glucosamine. Chitin is made up of a linear chain of acetylglucosamine groups, while chitosan is obtained by removing enough acetyl groups (CH_3_-CO) for the molecule to be soluble in most diluted acids [6]. Due to its biological characteristics of biocompatibility, biodegradability, and non-toxicity, chitosan is frequently employed as a biomaterial. It contains antibacterial and antifungal properties that make it useful as a medicinal agent and make it attractive for use in agriculture, medicine, the environment, the food and cosmetics industries, and textiles [7,8,9]. The existing literature has extensively covered the preparation of chitin and chitosan from marine sources. In general, the published methodologies for obtaining these two substances can be classified into two types: chemical methods and biological methods. In both cases, a preliminary stage of sample preparation is carried out wherein crustacean exoskeletons are mechanically separated and ground to powder. In addition, both methods require chemical extraction procedures (Figure 1).

Gelatin, on the other hand, results from the partial denaturation of collagen, the main structural protein in connective tissue, rendering a more soluble material with wider applicability [11]. Gelatin is a colloidal protein with unique characteristics. Much of its value lies in its inherent ability to form thermo-reversible gels when combined with water, a property that offers it a functionality that makes it a very versatile polymer for multiple industries, such as pharmaceuticals, cosmetics, and foods [12]. Gelatin properties depend on several factors, such as animal species, age, source tissues, and extraction conditions [13]. In fish, the skin provides a higher yield of and higher-quality gelatin than bone [14], and therefore, skin byproducts from filleting may represent a good source of this material [15].

The processing method is a crucial aspect of the manufacture of gelatin. Gelatin is divided into two types: type A (obtained under acidic conditions) and type B (obtained under alkaline conditions). A higher yield is obtained when the extraction process is carried out with acid solutions, since the acid converts the triple helix of collagen into single chains, while the alkaline solutions transform it into double chains [12]. In addition, the method used to remove moisture is fundamental to obtaining gels with good mechanical properties, which is also key in industrial scale-up due to the high economic costs it generates [16]. 

Despite the progress in polymer science, it is still a challenge to obtain natural polymers with good mechanical properties that allow for an extension of their range of applications according to their antimicrobial properties, nontoxicity, and biocompatibility. Therefore, the present work aims to evaluate different ways of extracting fish gelatin and chitosan from fishery byproducts (tench skins and bones and shrimp shells), thus optimizing extraction conditions, and to explore their behavior as active biopolymers with antimicrobial capabilities.

## 2. Results and Discussion

### 2.1. Chitosan Extraction and Quality

Table 1 shows the characterization of the starting residue for the extraction of chitosan (crustacean exoskeleton). The chemical composition of the prepared chitosan from prawn shells reached a yield of 20.45%, which was higher than that reported by other researchers [17], who reported a 12.93% yield from shrimp waste. However, yields of 28.4% for prawns, 23% for crabs, and 15.7% for lobsters, using a similar process for demineralization, were reported [18].

The lack of characterization in other studies makes comparison difficult. However, taking into consideration the value of prawns and assuming that the differences in starting material are not affected by the origin or other variables associated with the demographic origin of the product, the lower yield can be explained by the depolymerization of the chitosan polymer, loss of sample mass/weight due to the excessive removal of acetyl groups from the polymer during deacetylation, and loss of chitosan during extraction.

On the other hand, the ash content reached 1.17%, which indicates the efficiency of salt removal, as well as the conclusion that the ash content in high-quality chitosan must not increase beyond 1%. As reported by other researchers [18], although they had a higher ash percentage (26.3%) than that shown in this study (18.31%), it is possible to reduce the ash percentage to below 1% with the optimal treatment. Therefore, at this point, we find a clear point of improvement for future studies.

The great capacity of chitosan to absorb moisture from the environment makes it difficult to compare with other studies because the time, environment, and methodology, among other factors, distort the value of this parameter. Table 2 shows that the moisture content was 6.24%, which is lower than that obtained in a similar study [17].

The yield of the degree of deacetylation of the chitosan was 69.25%, which is at the lower limit of the yields that can be categorized as good [18,19,20].

The effect of antimicrobial activity was assessed through a microbiological quality test according to the ISO Standards. As shown in Table 3, none of the tested microorganisms showed any proliferation. According to other researchers [21], the positive charge of the chitosan is attributed to the presence of amino groups in the C5 position of their constituent monomers. Their positive charge induces interaction with negative charges on the membranes of microorganisms, altering the usual course of microbial homeostasis, preventing microbiological growth, and conferring bacteriostatic properties.

### 2.2. Fish Gelatin Extraction and Quality

Table 4 shows the yields obtained from the different tench residues. The chemical composition of fish gelatin prepared from the skin had a yield that reached 11.82%, which was lower than the results obtained by other researchers [12], who reported an 18.52% yield from skin waste in the best extractive conditions. The 40% decrease in yield may be due to a less careful separation of the skin from the muscle compared to the abovementioned studies and a worse thermal treatment, which, as the study itself explains through response surface models, is one of the factors that most alters the extraction process.

On the other hand, the chemical composition of fish gelatin prepared from spines had a yield that reached 2.31%, which was lower than that reported for milkfish (10.48%) [22]. The different bone compositions between the species tested, as well as the less aggressive acid treatment, may be the cause of the four-times-lower yields.

The mechanical properties are still poor compared to gels extracted from warm-blooded animals, mainly in terms of rheological properties. This may be due to the variability of electrolytes present in the animal, as well as the unique composition of the amino acid profile of hydroxyproline, an amino acid that, through its ability to establish hydrogen bonds, alters the stability of the final gelatin obtained [23]. Therefore, the application of salts is seen as the most viable solution to improve the rheological behavior and bring the properties of fish gelatin closer to those of gelatin extracted from warm-blooded animals.

**Figure 2:**
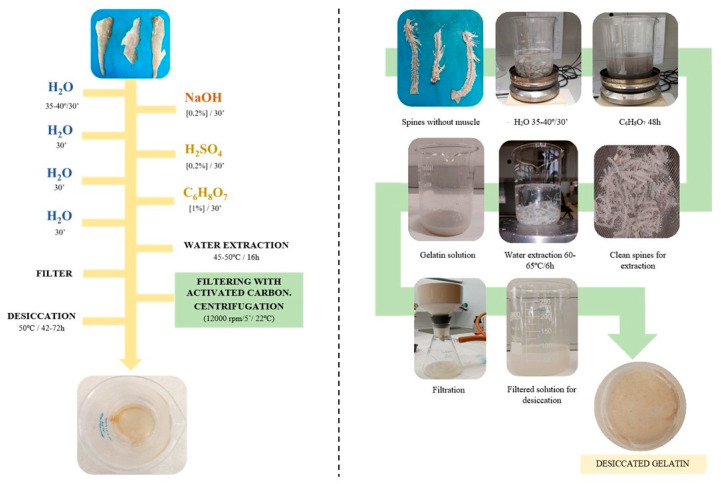
Overall process for preparation of fish gelatin from tench byproducts.

Process 1 in Figure 2 clearly shows fish gelatin with poor organoleptic properties. In this process, it is necessary to include extractive stages that deodorize and improve the sensory qualities of the final gelatin [16]. Process 2 in Figure 2 shows that the use of activated carbon in the filtering stage substantially improves the sensory aspect.

**Figure 3:**
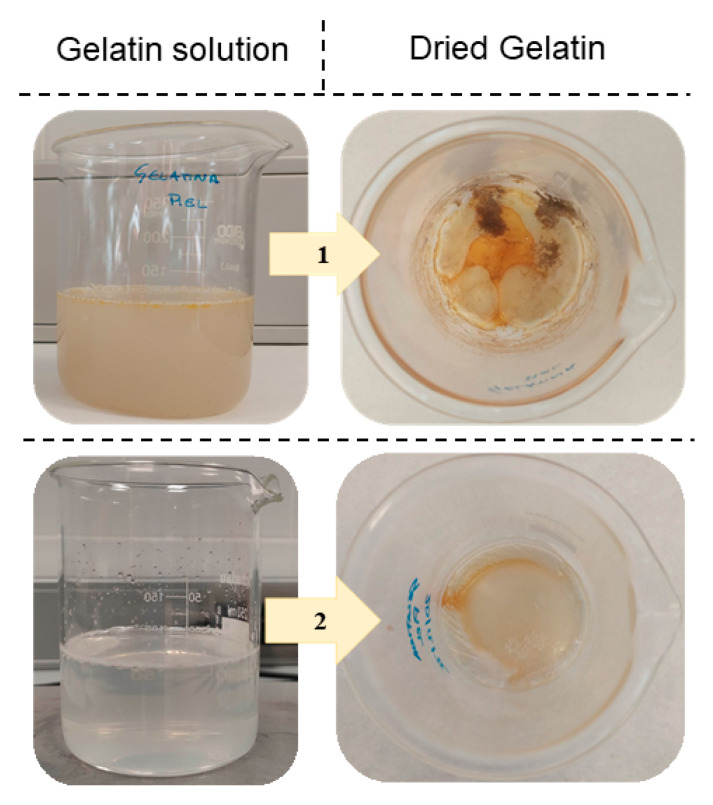
Result of applying an activated carbon filtration step in the production of fish gelatin (2) and without filter (1).

On the other hand, Figure 3 shows the difference between dried gelatin obtained without activated carbon filter (1) and with activated carbon filtering (2). Although it is true, as can be seen in Table 4, that the extraction yield decreases, obtaining gelatin with fewer impurities can increase its degree of acceptance and, therefore, its inclusion in the industry.

As is the case with most industrial processes, the transfer of the process in question is limited by the economic costs associated with obtaining the desired product. Through the present research, simple extraction conditions were achieved, with good yields, which allows chitosan and fish gelatin to be obtained from fish waste while making use of extraction techniques that are used extensively in the current industrial scene and, therefore, can be implemented with low economic costs, facilitating the transfer and inclusion of the extraction processes in the existing industrial framework that is already present.

### 2.3. Biopolymer Films and Antimicrobial Activity

The polymer formulation shares the plasticizers used, namely, lactic acid and glycerol. The presence of the plasticizers lactic acid and glycerol promotes improvement in the degree of swelling of the polymers [24], a consequence of the increase in the free volume [25]. In addition, lactic acid, in turn, decreases the availability of chitosan hydroxyl groups and thus the hydrophobicity of the polymers and their solubility in water, so that correct evaporation of the solvent generates a solvent–air interface, causing roughness on the surface, which can enable interactions with microorganisms or cells, favoring cell adhesion processes [26].

The excellent properties of chitosan, in terms of its antibacterial properties and low toxicity to animal cells, are widely documented [7,8,9]. However, its poor mechanical behavior has also been described, although in its favor, it has many free functional groups, which can be modified to increase its potential. These groups allow it to increase its degree of crosslinking and thus modify its amorphous–crystalline region ratio, or, by decreasing the presence of free hydroxyl groups in its structure and thus its hydrophobicity, make it ideal for coatings. The inclusion of the plasticizers improves the roughness and the degree of swelling, extending the range of applications with respect to those obtained by the polymers individually.

**Figure 4:**
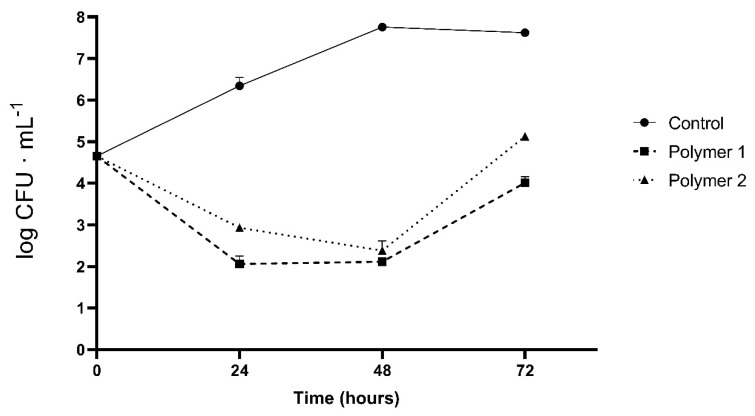
Antibacterial activity of polymers against *Escherichia coli* over 72 h of incubation. Control: growth without polymer. Polymer 1: polymer formulated with chitosan only. Polymer 2: polymer formulated with chitosan and fish gelatin. The results are expressed as log CFU·ml.

**Figure 5:**
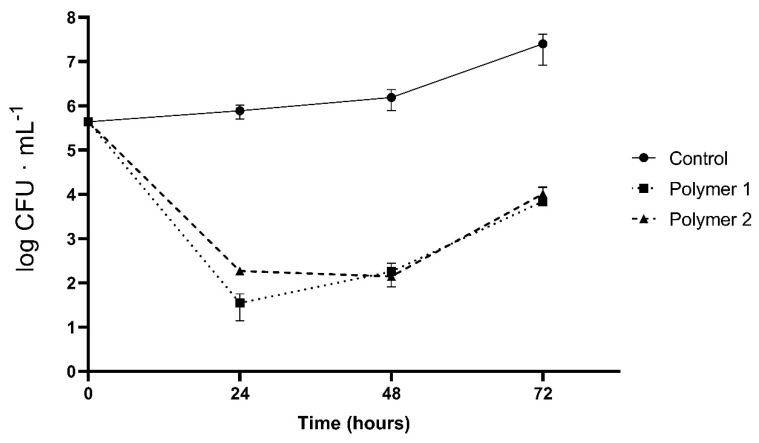
Antibacterial activity of polymers against *Listeria innocua* over 72 h of incubation. Control: growth without polymer. Polymer 1: polymer formulated with chitosan only. Polymer 2: polymer formulated with chitosan and fish gelatin. The results are expressed as log CFU·ml^−1^.

As shown in Figure 4 and Figure 5, the behavior of the polymer formed by fish gelatin and chitosan demonstrates worse antimicrobial properties. This behavior is expected, since the proportion of chitosan, a polymer with antimicrobial activity, is lower in the polymeric mixture.

Similar behavior was observed for both polymers and against both bacteria. In the first few hours, a bactericidal effect was observed; as suggested by other researchers, the adhesion of chitosan to the cell wall of the bacteria plays a fundamental role in breaking cellular homeostasis and forcing cell death [21]. However, with the passage of time, both bacteria can proliferate. This may be due to the lack of chitosan macromonomers in the medium as well as a possible bacterial adaptation that serves to evade the effects of chitosan. Therefore, the activity of chitosan is diluted, and it begins to rebound until it reaches the initial bacterial population. It clearly does have bactericidal but not bacteriostatic potential, since once a temporary threshold is reached (24 h), the growth curve of the microorganisms rises steadily.

## 3. Conclusions

As is the case with most industrial processes, the transfer of fish waste is limited by the economic costs associated with obtaining the desired product. Through the present research, simple extraction conditions were achieved with good yields, which allow chitosan and fish gelatin to be obtained from fish waste, making use of extractive techniques that are widely used in the current industrial scene and, therefore, can be implemented with low economic costs, facilitating the transfer and inclusion of extraction processes in the industrial framework that is already present.

Although more exhaustive characterizations of the obtained biopolymers are necessary, the polymeric mixture obtained by joining fish gelatin and chitosan shows the following:

It maintains, at least qualitatively, the mechanical properties of fish gelatin and introduces the excellent antimicrobial properties present in chitosan polymers.

The inclusion of the plasticizers improves the roughness and the degree of swelling, extending the range of applications with respect to the ranges obtained by the polymers individually.

The research carried out shows that there is a real and sustainable alternative that can be used to revalorize the high amount of waste generated by the fishing industry. Processes that enable the transformation of waste into high-added-value products applicable to a multitude of industrial sectors avoid the problems associated with waste and favor, among other things, the circularization of the fishing industry through efficient, sustainable processes that protect the environment around us.

## 4. Materials and Methods

### 4.1. Raw Material and Chemicals

The raw material used in this research included tench (*Tinca tinca*) bone and skin collected from fish farms in Badajoz, Spain. Frozen prawn shells (*Dendrobranchiata* spp.) were provided by Vegas del Guadiana Aquaculture Centre (Villafranco del Guadiana, Badajoz, Spain).

Citric acid, lactic acid, and glycerol were purchased from Panreac (pharma grade) (Castellar del Vallès, Spain). Hydrochloric acid and sodium hydroxide were obtained from Sigma-Aldrich (analytical grade) (Steinheim, Germany). Chromogenic Listeria Agar (Oxoid CM1084), Chromocult^®^ Coliform Agar (Merck, Darmstadt, Germany), chitosan (Sigma Aldrich, Saint Louis, MO, USA), and fish gelatin were obtained from Sosa Ingredients S.L. (Barcelona, Spain).

### 4.2. Chitosan Extraction

**Figure 6:**
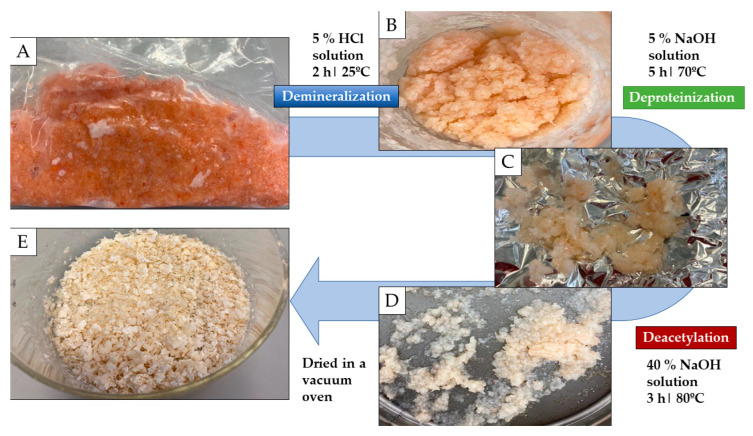
Chemical chitosan extraction. (**A**) Crushed exoskeletons. (**B**) Crushed exoskeletons after acid treatment. (**C**) Residue after diluted soda treatment. (**D**) Residue after concentrated soda treatment. (**E**) Chitosan flakes.

Figure 6 shows the chitosan extraction process. The exoskeletons were mechanically separated from the crustaceans and frozen at −20 °C. The frozen exoskeletons were crushed, using a mortar and pestle, into a homogeneous particle size. The crushed material was treated with a 5% HCl solution (*w*/*v* 1:10). The mixture was stirred for 2 h at room temperature, and then the supernatant was sieved and discarded.

The demineralized sample was mixed and treated with a 5% NaOH solution (*w*/*v* 1:10) for 5 h at 70 °C with magnetic agitation. After the established time, the chitin, obtained as free of impurities, was sieved and the supernatant was again discarded.

Chemical deacetylation was achieved by treating the extracted chitin with a 40% NaOH solution (*w*/*v* 1:10), stirred for 3 h at 80 °C with magnetic agitation. After the reaction, the material produced was filtered, and the solid was dried in a vacuum oven until the water was eliminated, leaving a final product with a powder-flake texture.

### 4.3. Chitin and Chitosan Characterization

The total nitrogen content was determined by the Kjeldahl method according to the standard procedures of AOAC.

The fat content in the solid residues was determined using the Soxhlet technique, quantifying the extractable substances with petroleum ether and considering the difference in weight, and was then expressed as a percentage with respect to the initial weight of the residue.

The amount of ash in the solid residues was determined by considering the difference in weight and was then expressed as a percentage with respect to the initial weight of the residue.

The percentage of soluble chitin was determined by dissolving 0.1 g of chitin in 25 mL of a solution of N, N-dimethylacetamide (DMAc) with 5% lithium chloride (LiCl), for 72 h at 25 °C with constant agitation. Subsequently, it was filtered through a nylon membrane, and the samples were dried at 100 °C for 24 h. The amount of dissolved chitin was calculated from the weight difference.

The amount of moisture in the shredded residues was determined by considering the difference in weight and was then expressed as a percentage with respect to the initial weight of the residue.

The percentage of soluble chitosan was determined by dissolving 0.1 g in 25 mL of a 0.1 M acetic acid solution for 72 h at 25 °C with constant agitation; it was then filtered through cellulose filter paper (0.45 µm), and the samples were dried at 100 °C for 24 h. The amount of dissolved chitosan was calculated using the weight difference.

The degree of deacetylation (DA) was determined by using the carbon/nitrogen (C/N) ratio obtained through elemental analysis (Equation (1)).

(1) $$\mathrm{DA}=\left(\frac{\frac{C}{N}-5.145}{6.861-5.145}\right)\times 100$$

The value of 5.145 represents the completely N-deacetylated chitosan (C_6_H_11_O_4_N repeat unit), and 6.186 is the fully N-acetylated polymer (C_8_H_13_O_5_N repeat unit) [27].

The detection and calculation of the following microorganisms were carried out by applying the ISO Standards established in the applicable regulations: aerobic mesophilic microorganisms (UNE-EN ISO 4833-1:2014), total coliforms (ISO 4832:2006), *Escherichia coli* (ISO 16649-2:2001), molds and yeasts (ISO 21527-2:2008), *Staphylococcus aureus* (UNE-EN ISO 6888-1:2022), *Clostridium perfringens* (UNE-EN ISO 7937:2005), *Salmonella* spp. (UNE-EN ISO 6579-1:2017), and *Listeria monocytogenes* (UNE-EN ISO 11290-1:2018).

### 4.4. Gelatin Extraction from Spines and Skin

Gelatin extraction from the spines was completed with some modifications [22]. The bones were soaked in a 9% citric acid solution (*w*/*v*), where the ratio of bones to acid was 1:3 (the fish bones were soaked in a citric acid solution with a volume of 3 times the weight of the fish bones) for 48 h. Subsequently, the bones were washed with flowing water to remove the acid residue until the pH value was between 5 and 6. The acquired soft bones (ossein) were extracted using distilled water at 60–65 °C for six hours with mild stirring. 

The obtained gelatin solution was filtered using a filter cloth and was then dried in the cabinet dryer at a temperature of 50 °C for 48–72 h.

Gelatin extraction from the skin was completed with some modifications [12]. The clean skins were soaked in NaOH (0.2%) (4:1 *w*/*v*) for thirty minutes, with mild stirring, at 22 °C, followed by washing in distilled water. Subsequently, the skins were soaked in H_2_SO_4_ (0.2%) (4:1 *w*/*v*) for thirty minutes, with mild stirring, at 22 °C, followed by washing in distilled water. Then, the skins were soaked in C_6_H_8_O_7_ (1%) (4:1 *w*/*v*) for thirty minutes, with mild stirring, at 22 °C, and the skins were then washed in distilled water. The acquired skins were extracted using distilled water at 45–50 °C for sixteen hours with mild stirring. The obtained gelatin was filtered twice through a Bunsen funnel, the second time using activated carbon. Subsequently, it was centrifuged at 12,000 rpm for 5 min at 22 °C. Finally, the sample was dried in the cabinet dryer at a temperature of 50 °C for 48–72 h.

### 4.5. Gelatin Fish Characterization

The yield of gelatin extraction was calculated by considering the wet weight of the skins/spines before extraction and the dry weight of the gelatin by using the following equation (Equation (2)):

(2) $$\mathrm{Yield}\;\mathrm{of}\;\mathrm{Gelatin}\;\mathrm{extraction}\;\left(\%\right)=\frac{\mathrm{Weight}\;\mathrm{of}\;\mathrm{dried}\;\mathrm{gelatin}\;\left(g\right)}{\mathrm{Weight}\;\mathrm{of}\;\mathrm{wet}\;\mathrm{skin}/\mathrm{spines}\;\left(g\right)}\times 100$$

### 4.6. Biopolymer Synthesis

Two film-forming solutions were prepared. Polymer 1: commercial chitosan (Sigma Aldrich) (2% *w*/*v*) was dissolved in a 1% lactic acid solution until complete solubilization. Afterwards, glycerol was added to the solution at 50% (*w*/*w*). The dissolution was stirred in an ultrasonic bath (P-Selecta, mod516, Barcelona, Spain) until solubilization. Polymer 2: commercial chitosan (Sigma Aldrich) and fish gelatin (Sosa Ingredients S.L.) (1% and 3%, respectively; *w*/*v*) were dissolved in a 2% lactic acid solution until complete solubilization. Afterwards, glycerol was added to the solution at 50% (*w*/*w*). The dissolution was stirred in an ultrasonic bath (P-Selecta, mod516, Barcelona, Spain) until solubilization. The obtained solutions were poured into polystyrene Petri dishes Ø 150 mm (50 g/dish) and left to dry at room temperature in the dark for 24–48 h until film formation. The same formulation was also prepared without fish gelatin.

### 4.7. Antimicrobial Activity

The bacteria strains used in the antimicrobial experiment were obtained from the Spanish Type Culture Collection (CECT) of Valencia University: *Listeria innocua* (CECT 910) and *Escherichia coli* (CECT 45).

An adaptation of the broth dilution method was used to study the antimicrobial activity of the biopolymers. Falcons were prepared with 10 mL of Muller–Hinton broth. Then, 1.5 × 1.5 cm pieces of each biopolymer were cut and introduced into the prepared medium. Finally, the preparations were inoculated with *Listeria innocua* and *Escherichia coli* (~10^3^ CFU·mL^−1^). 

Counts of the microorganisms were carried out at 0, 24, 48, and 72 h on a plate of Chromogenic Listeria Agar (Oxoid CM1084) for *L. innocua* and Chromocult^®^ Coliform Agar (Merck, Darmstadt, Germany) for *E. coli.* The plates were incubated at 37 °C with readings at 24–48 h. The results were expressed as log CFU·ml^−1^.

## Figures and Tables

**Table 1:** Characterization of the initial waste (crustacean exoskeletons).

Characteristics	Composition (%)	SD (%)
Protein	31.45	±2.31
Total Fat	13.30	±1.28
Ash	18.31	±1.17
Chitin	35.89	±3.96

**Table 2:** Percentage yield from the chitosan extraction process.

Characteristics	Composition (%)	SD (%)
Ash	1.17	±0.41
Moisture	6.24	±1.62
Chitosan yield from crustacean exoskeleton chitin	20.45	±3.91
Degree of deacetylation of chitosan	69.25	±4.23

**Table 3:** Microbiological quality test of the chitosan extraction process.

Microorganisms							
Total coliform	*E. coli*	Molds and yeast	Aerobic mesophilic	*S. aureus*	*C. perfringens*	*Salmonella* spp.	*L. monocytogenes*
<1	<1	<1	<1	<1	<1	Absence	Absence

**Table 4:** Extraction yield of fish gelatin.

Raw Material	Yield
Spine	2.31%
Skin	11.82%
Skin (filtrated with active carbon)	7.11%
